# Combined metabolomics and transcriptomics analysis of rats under neuropathic pain and pain-related depression

**DOI:** 10.3389/fphar.2023.1320419

**Published:** 2023-12-08

**Authors:** Caiyun Xi, Liqiong He, Zhifeng Huang, Jianxi Zhang, Kailu Zou, Qulian Guo, Changsheng Huang

**Affiliations:** ^1^ Department of Anesthesiology, Xiangya Hospital, Central South University, Changsha, China; ^2^ National Clinical Research Center for Geriatric Disorders, Xiangya Hospital, Central South University, Changsha, China

**Keywords:** neuropathic pain, depression, metabolomics, transcriptomics, anterior cingulate cortex

## Abstract

Neuropathic pain often leads to negative emotions, which in turn can enhance the sensation of pain. This study aimed to investigate the molecular mechanisms mediating neuropathic pain and negative emotions. Chronic constriction injury (CCI) rats were used as model animals and behavioral tests were conducted to assess pain and negative emotions. Then, the rat anterior cingulate cortex (ACC) was analyzed using UPLC-MS/MS and subsequently integrated with our previously published transcriptome data. Metabolomics analysis revealed that 68 differentially expressed metabolites (DEMs) were identified, mainly in amino acid metabolites and fatty acyls. Combined with our previously published transcriptome data, we predicted two genes that potentially exhibited associations with these metabolites, respectively Apolipoprotein L domain containing 1 (Apold1) and WAP four-disulfide core domain 1 (Wfdc1). Taken together, our results indicated that peripheral nerve injury contributing to neuropathic pain and pain-related depression may be associated with these metabolites and genes. This research provides new insights into the molecular regulatory mechanism, which could serve as a reference for the treatment of neuropathic pain and pain-related depression.

## 1 Introduction

Epidemiological research suggests that neuropathic pain is prevalent in approximately 7%–10% of the general population, significantly impacting individuals’ physical and mental wellbeing (Colloca et al., 2017). The coexistence of neuropathic pain with psychiatric disorders renders it less responsive to conventional therapeutic approaches (Simon et al., 1999; McWilliams et al., 2004). The presence of abnormal psychiatric conditions may exacerbate the duration and severity of pain, perpetuating a detrimental cycle of pain and emotional distress (Goyal et al., 2014; Gilam et al., 2020). Consequently, it is imperative to find novel and efficacious interventions for neuropathic pain and negative emotions.

It is noteworthy that the anterior cingulate cortex (ACC) has gained significant recognition as a region that effectively processes pain signals both physiologically and pathologically (Zhuo, 2008; Barthas et al., 2015; Kummer et al., 2020). Its rostral part plays important roles in pain-induced negative emotions, while the caudal part is primarily involved in the perception of pain (Zhuo, 2008; Kummer et al., 2020). ACC modulates pain and negative emotions through mechanisms including activation of microglia (Duan et al., 2022), altered synaptic plasticity (Chen et al., 2020), and reduction of extracellular matrix protein (Li et al., 2021). Activation of ACC leads to pain unpleasantness (Tang et al., 2005), while inhibition of ACC could reduce chronic pain and unpleasantness (Vogt, 2005; Zhuo, 2007; Zhuo, 2014). We have previously published transcriptome analysis of neuropathic pain in the ACC after nerve injury and found interesting gene changes in this region (Zhang et al., 2022). Therefore, our next research on ACC will further contribute to the pathogenesis of neuropathic pain and negative emotions, as well as offer viable therapeutic alternatives.

The brain is the most metabolically active organ in the body. Previous researches have established a strong correlation between the occurrence of neuropathic pain and abnormalities in central nervous system metabolites including glutamate, glutamine (Zunhammer et al., 2016; Archibald et al., 2020) and tetrahydrobiopterin (Latremoliere et al., 2015). Abnormal changes in these metabolites have also been associated with negative emotions such as depression. For example, the cerebrospinal fluid of a macaque model of depression exhibited metabolites that were predominantly marked by disruptions in fatty acyls, biosynthesis, ABC transport systems, and amino acid metabolism (Deng et al., 2019). However, the relationship between changes in metabolites in the ACC and neuropathic pain as well as negative emotions remains unclear.

Transcriptomics can help scientists study the effect of diverse biomarkers and identify their mechanisms. Metabolomics, on the other hand, enables the identification of comprehensive metabolic pathways that occur during particular pathological and physiological processes (Johnson et al., 2016). The integration of metabolomics and transcriptomics has emerged as a potent approach for providing novel insights into pivotal genes and metabolites from the complex regulatory networks of multisystem diseases. This methodology has been widely used in neurological diseases (Horgusluoglu et al., 2022; Wang et al., 2023). Nevertheless, there are no studies combining metabolomics and transcriptomics analysis to unravel the underlying mechanisms of neuropathic pain and negative emotions.

The main purpose of this study was to explore the occurrence of neuropathic pain and pain-related depression in rats after the implementation of the chronic constriction injury (CCI) model, as well as to investigate potential molecular mechanisms associated with these conditions through metabolomic and transcriptomic methodologies. Researchers may be able to apply these findings to the diagnosis and treatment of neuropathic pain and pain-related depression in the future.

## 2 Methods

### 2.1 Animals

Adult male Sprague-Dawley rats (Hunan SLAC Laboratory Animal Co., Ltd., Changsha, China), weighing 220g–250 g, were utilized for experiments. Six rats were housed per cage in a temperature-controlled environment (25°C–28°C) with a 12-h light/dark cycle. We obtained approval from the Institutional Ethics Committee of Xiangya Hospital for all procedures, and performed experiments according to the National Institutes of Health Guidelines for Laboratory Animal Care and Ethical Guidelines.

### 2.2 CCI model

The CCI model was established using the method of Bennett and Xie (Bennett and Xie, 1988). Expose the sciatic nerve by cutting the left thigh and rats were anesthetized with pentobarbitone sodium. Four knots were tied at 1-mm intervals on the main nerve trunk using a 4–0 chromic gut wire. The sciatic nerve was ligated with enough force to cause transient twitching of the surrounding muscles and mild compression of the sciatic nerve membrane, without affecting the nerve’s blood supply. A ligation of the nerve was performed with the same tightness. In the sham group, the left sciatic nerve was exposed without ligation.

### 2.3 Behavioral assessment

All behavioral tests were conducted in the following order: paw withdrawal mechanical threshold (PWMT), paw withdrawal thermal latency (PWTL), sucrose preference test (SPT) and forced swimming test (FST).

PWMT was assessed on 1 day before CCI surgery and 3, 7, 14, 21 days after CCI surgery (*n* = 8). Measurements on 1 day before CCI surgery were used as the baseline. The rats were stimulated vertically with Von Frey filaments (North Coast Medical, San Jose, CA, United States) (Shen et al., 2019) on the mid-foot of the left hind limb, starting from 0.4 g and reaching a maximum of 15 g. PWMT of the rats was calculated using the UP AND DOWN method (Shen et al., 2019).

PWTL was assessed using a thermal pain tester (Tes7370, Ugo Basile, Comerio, Italy) (Porsolt et al., 1978; Shen et al., 2019) (*n* = 8). Continuous thermal stimulation was applied to the plantar surface of the left hind limb after the rats were accustomed to the cage for 30 min. When the rats lifted or licked the paw, timer and radiant heat were automatically shut off. The mean of the three latency periods was taken to obtain the PWTL.

SPT was assessed on 1 day before CCI surgery and 7, 14, 21 days after CCI surgery (*n* = 8). SPT is a good indicator of depression-like behavior as it reflects the lack of interest in the animal. According to a previous study (Wang et al., 2008), the rats were acclimatized to sugar water for 3–5 days before the test. The rats were housed in a single cage and given a weighed bottle of 1% sugar water and a bottle of plain water. To avoid the effect of water position, the positions were swapped once after 12 h. The quality of the sugar water and pure water was measured after 24 h.

FST is a behavioral despair test that assesses animals’ depressive state based on the level of despair (*n* = 8). Freshwater (24°C ± 1°C) was poured into a clear cylinder for the test, which was enough to submerge animals. We recorded the entire 5-min session and calculated the duration of immobility in the last 3 min offline. The definition of despair behavior was to float without making any movements other than keeping one’s nose above water.

### 2.4 RNA sequence and data analysis

Both sides of ACC were dissected under RNase-free conditions. Shanghai Biotechnology Corporation prepared the cDNA library from all ACC samples and performed the sequencing. The expression of annotated genes was analyzed by calculating fragments per kilobase of transcript per million mapped reads (FPKM) (Mortazavi et al., 2008). Fold change (FC) was calculated based on the FPKM value.

### 2.5 Extraction of metabolites and metabolome profiling

Steel beads were added to homogenize the ACC (20mg) samples for 20 s using a ball mill (30HZ). The samples were then centrifuged at 3,000 r/min for 30 s at 4°C. Next, 400 μL of 70% methanolic water internal standard extract was added, and the samples were centrifuged at 12,000 rpm for 30 min at 4°C. The resulting supernatants were transferred to vials for analysis. The samples were analyzed using ultra-performance liquid chromatography (UPLC, SCIEX, United Kingdom) coupled with tandem mass spectrometry (MS/MS, SCIEX, United Kingdom). An ACQUITY UPLC T3 column was used to separate the samples. Detection was performed using electrospray ionization.

### 2.6 Data processing of untargeted metabolomics

To further analyze the raw data, Proteowizard (http://proteowizard.sourceforge.net/) was used to convert it to mzXML.After pareto-scaling preprocessing, multivariate data analysis was performed, including quality control (QC), principal component analysis (PCA), orthogonal partial least squares-discriminant analysis (OPLS-DA), and univariate statistical analysis, such as FC analysis and t-tests. Calculations of FC values were based on the mean concentrations of metabolites in each group. The screening criteria for selecting differentially expressed metabolites was variable importance in projection (VIP) > 1 and *p* < 0.05. Analyzing the interactions among differentially expressed ACC metabolites was carried out using Ingenuity pathway analysis (IPA) (www.ingenuity.com). In brief, an interaction network with a score >2 was generated using IPA software.

### 2.7 Combined metabolome and transcriptome analysis

Differentially expressed metabolites (DEMs) were obtained from metabolome data and differentially expressed genes (DEGs) from transcriptome data based on metabolite content and gene expression data. All DEMs and DEGs were determined by comparisons between two different groups, each with at least three biological replicates. Association analysis was performed for metabolites and genes.

### 2.8 Real-time quantitative polymerase chain reaction (RT-qPCR)

Under deep anesthesia, rats were sacrificed at different times. Following phosphate-buffered saline perfusion, ACC were tracted from each rat. RT-qPCR reagent mixes were prepared with the TransStart Tip Green qPCR SuperMix Kit (TransGen Biotech, Beijing, China) and performed using an ABI Prism 7300 PCR system (Applied Biosystems). The relative expression of the target gene compared to the housekeeping gene ACTIN was calculated by 2^−ΔΔCt^ method. All primers were obtained from Shanghai Biotechnology Company and the primer sequences are listed in Table 1.

**TABLE 1:** The primer sequences of RT-qPCR.

Primer	Forward	Reverse
β-actin	5′-CATCCTGCGTCTGGAACCTGG -3′	5′-TAATGTCACGCACGATTTCC-3′
Apold1	5′-GCGAACTCCTGAGCTGCCTTG-3′	5′-AGCCGAAGAAGACGATGAAGTAGAC-3′
Arc	5′-CATCTGTTGACCGAAGTGTCCAAG-3′	5′-AGCCGTCCAAGTTGTTCTCCAG-3′
Dbp	5′-ACCGCTTCTCAGAGGAGGAATTG-3′	5′-TTGGCTGCTTCATTGTTCTTGTACC-3′
Igkc	5′-CAAAGGTCCTGAGGTGCCAC-3′	5′-GGAGGGAAGATTGGAAGGAGC-3′
Klf4	5′-TCCACCTAAGCCCAAACCTTTCC-3′	5′-CATCACTGTCCTCATTCTCCTCCTC-3′
Lnpep	5′-CAGAACCAAATCCAGCAGCAGAC-3′	5′-CGCAGGCAAATTCCAGCAAGG-3′
Lrp2	5′-AGAATCTCAGGTGGTTCGCTATGG-3′	5′-GTTGCTTGCTGGCTTGGAAGAC-3′
Wfdc1	5′-CGGAGGAAGTGTTACAAGCAGAGG-3′	5′-ATAGCCTGAGGGACAGAGGAGTG-3′
Zdbf2	5′-GGAAGAAGAGCAGCAGGAAGAGG-3′	5′-GTCGGTTCAGATAGGGCACTCAG-3′

### 2.9 Statistical analysis

For behavioral and biochemical data, GraphPad Prism (GraphPad Software, San Diego, CA, United States)was used for statistical analysis. The significance of the differences between the two groups was analyzed using unpaired Student’s t-test, and multiple comparisons were performed by one-way or two-way analysis of variance (ANOVA) followed by Tukey’s multiple comparison test. *p* < 0.05 was considered statistically significant. Metabolome and transcriptome analysis have been detailed in Sections 2.6, 2.7.

## 3 Results

### 3.1 Behavioral characterization of rats after CCI surgery and QC analysis

**FIGURE 1:**
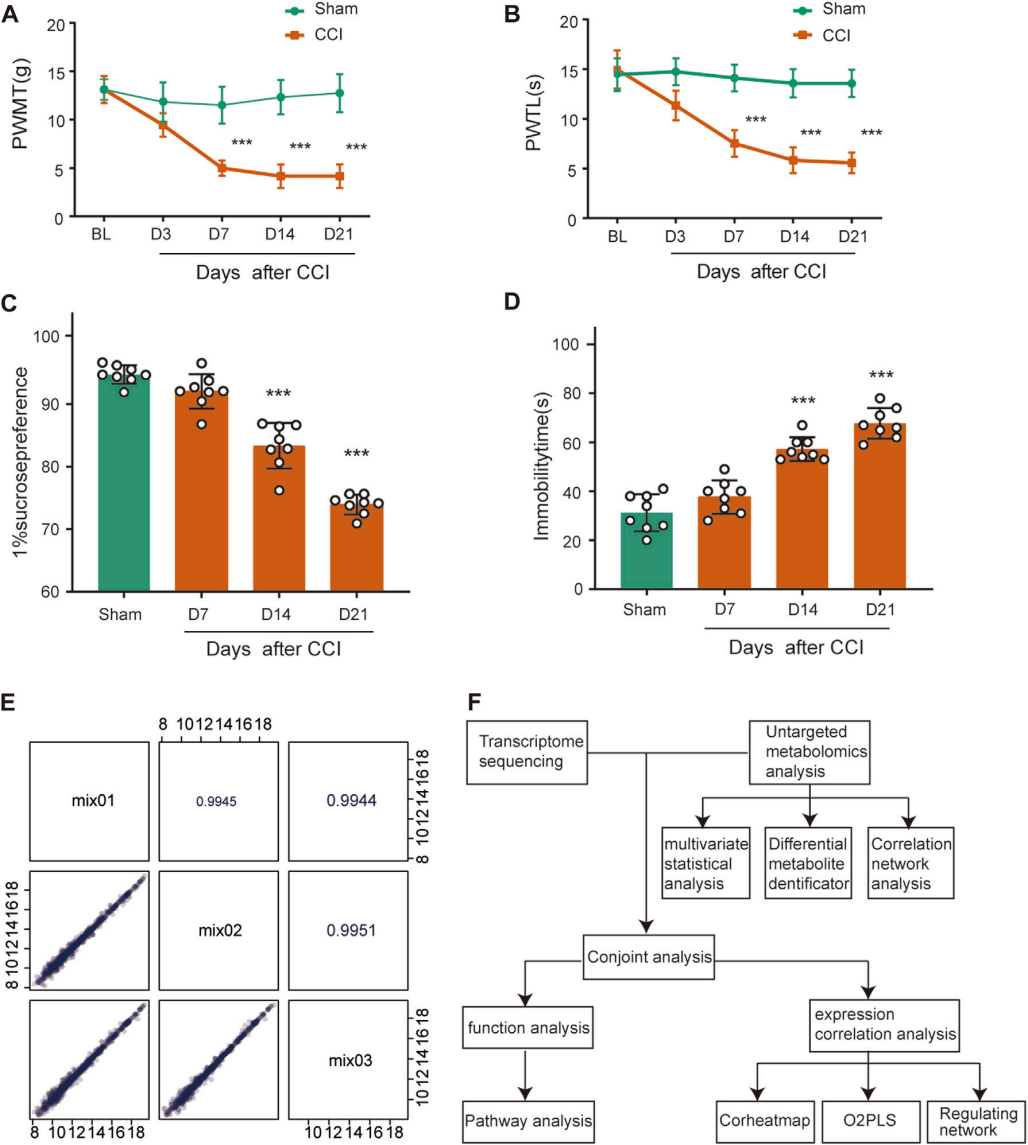
Behavioral characterization of rats following CCI surgery and QC analysis. **(A,B)** Mechanical allodynia and thermal hyperalgesia were induced after CCI surgery (*n* = 8). **(C)** Sucrose preference test. **(D)** Forced Swimming Test. ***p < 0.001, *n* = 8, one-way or two-way ANOVA followed by Tukey’s multiple comparison test. **(E)** Pearson correlation analysis for QC samples. The horizontal and vertical coordinates are the metabolite contents, and each point represents a metabolite. The upper-right diagonal square is the Pearson correlation coefficient of the corresponding QC samples. **(F)** The flowchart of combined metabolomics and transcriptomics analysis.

To determine the mechanical and thermal allodynia in rats, the PWMT and PWTL were assessed in the sham and CCI rats at 3, 7, 14 and 21 days postoperatively. The CCI group showed significantly lower ipsilateral PWMT and PWTL on postoperative day 7 compared to the sham group, and this difference persisted throughout the 21-day observation period (Figures 1A, B, *n* = 8, *p* < 0.001), suggesting that rats in the CCI group exhibited significant hyperalgesia.

SPT and FST were used as a measure of depression-like behavior. Rats at CCI 14 days and 21 days had less preference for sucrose compared with the sham group (Figure 1C, *n* = 8, *p* < 0.001), which showed anhedonia, a core symptom of depression. The sham and CCI rats were forced to swim for 5 min and immobility time in the last 3 min was recorded. The CCI 14 days and 21 days groups spent 26.0% and 36.5% less time immobile, respectively, than the sham group (Figure 1D, *n* = 8, *p* < 0.001), also indicating depressed emotion after peripheral nerve injury.

We then conducted QC analysis for the obtained ACC samples. A mixture of sample extracts was used to prepare QC samples that were used to test the reproducibility of analyzed samples processed in the same way. Pearson correlation analysis was performed on QC samples in Figure 1E. The diagonal squares represent the QC sample names, and the lower-left diagonal squares are scatter plots of the correlation of the corresponding QC samples, with the horizontal and vertical coordinates being the metabolite contents, and each point representing a metabolite. The upper-right diagonal square is the Pearson correlation coefficient of the corresponding QC samples, and the *R* ^2^ value for each QC sample is close to 1, which suggests that the experiment data is stable.

### 3.2 Statistical analysis of ACC samples

**FIGURE 2:**
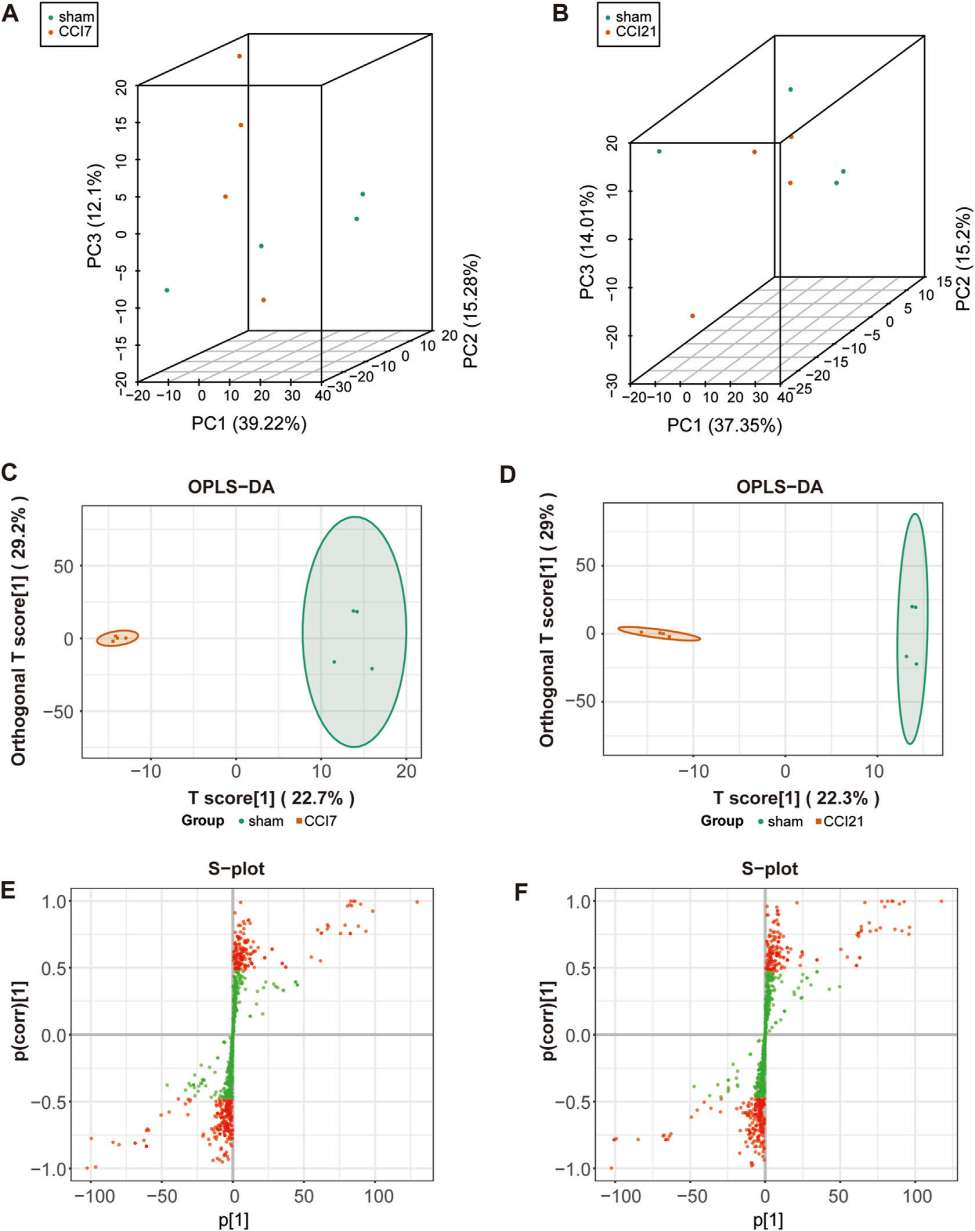
3D-PCA plot, OPLS-DA model and S-plot map of the rats following CCI surgery. **(A,B)** 3D-PCA plot showing CCI 7 days **(A)**/CCI 21d **(B)** (red) group and sham (green) group (*n* = 4 per each group). *Each point represents* a *sample*, and *the distance* between *the* points *represents the* magnitude *of the* feature difference between *the* samples. **(C,D)** OPLS-DA scores plot between CCI 7 days **(C)**/CCI 21d group **(D)** and sham group. **(E,F)** S-plot map between CCI 7 days **(E)**/CCI 21 days group **(F)** and sham group. The red dot means VIP >1.0.

In order to visualize the overall differences between individual group samples, unsupervised multivariate principal component analysis was used. The 3D-PCA plot (Figures 2A, B) displayed the results with the PC1 (x-axis), the PC2 (y-axis) and PC3 (z-axis) representing the scores of the first, second and third principal components, respectively. Slight differences in the samples will result in closer scattering points. The 3D-PCA plot proved an obvious metabolic differentiation between sham group and CCI 7 days/CCI 21 days group.

OPLS-DA model (Figures 2C, D) was used in the experiment to find potential markers by maximizing the differences and highlighting key variables. A successful distinction between CCI 7 days (R^2^X = 0.513, R^2^Y = 0.996 and Q^2^ = 0.609)/CCI 21 days (R^2^X = 0.519, R^2^Y = 0.993 and Q^2^ = 0.524) and the sham group was shown in OPLS-DA score plot. The horizontal coordinate indicated the predictive component scores and the horizontal direction showed the gap between the CCI 7 days/CCI 21 days group and sham group. The vertical coordinate indicated the orthogonal component scores and the vertical direction showed the gap within groups. The results showed that the sham group and CCI 7 days/CCI 21 days group could be well separated. The S-map (Figures 2E, F) was used to identify key metabolites that contribute to CCI 7 days, CCI 21 days and sham group differentiation. From the S-map, various metabolites were identified responsible for the separation between each pair of groups and were considered to be potential biomarkers.

### 3.3 Differential metabolite identification and pathway analysis

**FIGURE 3:**
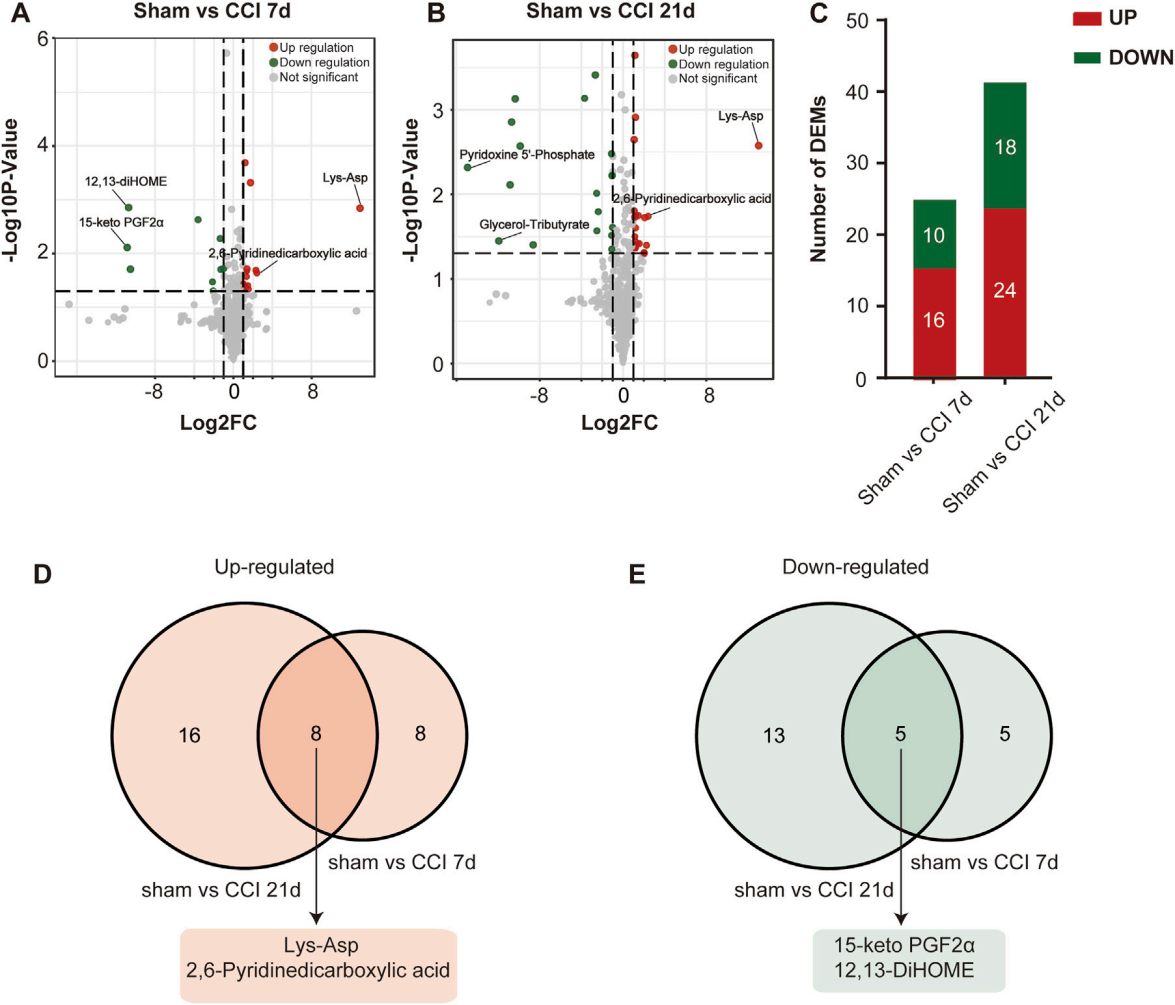
Differential metabolite identification. **(A,B)** Volcano plots of all the DEMs of the sham group compared with CCI 7 days **(A)** and 21 days **(B)** groups. Log_2_FC is plotted as the abscissa, and -log_10_ (*p*-value) is plotted as the ordinate. Upregulated genes are red and downregulated genes are green. Gray dots represent genes with no significant difference. **(C)** Histogram showing the statistics of up-and downregulated DEMs at CCI 7 days and CCI 21 days compared with the sham group. **(D,E)** The intersection of CCI 7 days and CCI 21 days upregulated **(D)** and downregulated **(E)** metabolites. The top two metabolites with the most significant changes in the intersection were listed separately.

The metabolites that were significantly different between the sham and CCI groups were screened (metabolites with VIP >1,∣log_2_FC∣>1 and *p* < 0.05 are generally considered to be significantly different) and listed in Tables 2, 3. Compared to the sham group, as many as 26 (16 up- and 10 downregulated) DEMs and 42 (24 up- and 18 downregulated) DEMs respectively occurred in CCI 7 days and CCI 21 days groups (Figures 3A–C). Metabolites with significant changes could be classified into amino acid metabolites, organic acid and its derivatives, benzene and substituted derivatives, carbohydrates and their metabolites, nucleotide and its metabolites, alcohol and amines, glycerol phospholipids, glycerol lipids, coenzyme and vitamins, heterocyclic compounds and fatty acyls, with amino acid metabolites being the largest group. Compared to the sham group, the most significantly upregulated metabolites of CCI 7 days and 21 days group were lysine-aspartic acid (Lys-Asp) and 2,6-pyridine dicarboxylic acid. The most significantly downregulated metabolites of CCI 7 days were 15-keto prostaglandin F2α (15-keto-PGF2α) and 12,13-dihydroxy-9Z-octadecenoic acid (12,13-diHOME). CCI 21 days group presented different results, showing that pyridoxine 5′-phosphate (PNP) and glycerol-tributyrate were most downregulated. We separately integrated the metabolites that were significantly up- and downregulated at CCI 7 days and CCI 21 days. Eight metabolites were significantly upregulated at both CCI 7 days and CCI 21 days, with Lys-Asp and 2,6-pyridine dicarboxylic acid being the most notable changes (Figure 3D). Five metabolites were significantly downregulated at both CCI 7 days and CCI 21 days, with 15-keto-PGF2α and 12,13-diHOME changed the most notably (Figure 3E).

**TABLE 2:** Significant metabolites differences in the rat ACC between the CCI 7d and sham groups.

Index	Compounds	Class	Log2FC	Type
MEDN2129	Lys-Asp	Amino acid and Its metabolites	12.942	up
MEDP2133	2,6-Pyridinedicarboxylic acid	Heterocyclic compounds	2.399	up
MEDP0272	5-Hydroxyindole-3-Acetic Acid	Heterocyclic compounds	2.304	up
MEDN1480	N-Arachidonoyl-L-Alanine	FA	1.747	up
MEDP2329	Lys-Val	Amino acid and Its metabolites	1.555	up
MEDN0621	Indoxylsulfuric acid	Heterocyclic compounds	1.462	up
MEDP1914	N-(1-Deoxy-1-fructosyl) phenylalanine	Amino acid and Its metabolites	1.382	up
MEDP2328	Asn-Trp	Amino acid and Its metabolites	1.382	up
MEDN0485	D-Fructose-1,6-Biphosphate-Trisodium Salt	Carbohydrates and Its metabolites	1.336	up
MEDN0321	Quinic acid	Organic acid And Its derivatives	1.236	up
MEDP0156	5-Methyluridine	Nucleotide And Its metabolites	1.185	up
MEDP1166	PC (16:0/2:0)	GP	1.106	up
MEDN0093	3-Hydroxyanthranilic Acid	Benzene and substituted derivatives	1.060	up
MEDN0852	3-Amino-4-Hydroxybenzoic Acid	Benzene and substituted derivatives	1.060	up
MEDN2067	3-Amino-5-hydroxybenzoic acid	Organic acid And Its derivatives	1.060	up
MEDP0177	Thymine	Nucleotide And Its metabolites	1.030	up
MEDP1196	Biotinamide	Alcohol and amines	−1.005	down
MEDP1441	Carnitine C5:1	FA	−1.283	down
MEDP1947	Ser-Ile	Amino acid and Its metabolites	−1.349	down
MEDP0734	2-Aminophenol	Benzene and substituted derivatives	−2.070	down
MEDP0791	4-Aminophenol	Benzene and substituted derivatives	−2.070	down
MEDP1177	N-Methyl-α-aminoisobutyric acid	Amino acid and Its metabolites	−2.138	down
MEDP2571	Asn-Ile	Amino acid and Its metabolites	−3.612	down
MEDN0249	Pantothenol	CoEnzyme and vitamins	−10.525	down
MEDN1081	12,13-DiHOME	FA	−10.709	down
MEDN1454	15-keto Prostaglandin F2α	FA	−10.855	down

**TABLE 3:** Significant metabolites differences in the rat brain between the CCI 21d and sham groups.

Index	Compounds	Class	Log_2_FC	Type
MEDN2129	Lys-Asp	Amino acid and Its metabolites	13.035	up
MEDP2133	2,6-Pyridinedicarboxylic acid	Heterocyclic compounds	2.417	up
MEDP1377	Carnitine C20:1-OH	FA	2.254	up
MEDN1162	p-Tolyl Sulfate	Organic acid And Its derivatives	2.086	up
MEDP2345	His-Val	Amino acid and Its metabolites	2.049	up
MEDP2576	Val-His	Amino acid and Its metabolites	2.049	up
MEDN0370	LPA (0:0/18:0)	GP	2.022	up
MEDP2265	2-Amino-3-phosphonopropionic-acid	Organic acid And Its derivatives	1.556	up
MEDP1901	Carnitine C9:1-OH	FA	1.471	up
MEDP2328	Asn-Trp	Amino acid and Its metabolites	1.380	up
MEDN0093	3-Hydroxyanthranilic Acid	Benzene and substituted derivatives	1.212	up
MEDN0852	3-Amino-4-Hydroxybenzoic Acid	Benzene and substituted derivatives	1.212	up
MEDN2067	3-Amino-5-hydroxybenzoic acid	Organic acid And Its derivatives	1.212	up
MEDN0621	Indoxylsulfuric acid	Heterocyclic compounds	1.211	up
MEDN0140	Xanthine	Nucleotide And Its metabolites	1.205	up
MEDN0321	Quinic acid	Organic acid And Its derivatives	1.193	up
MEDP1218	3-Amino-2-menacarboxylic-acid	Benzene and substituted derivatives	1.190	up
MEDN1006	Uric acid	Organic acid And Its derivatives	1.164	up
MEDP0147	1-Methylhistidine	Amino acid and Its metabolites	1.140	up
MEDN1092	4-acetoxyphenol	Benzene and substituted derivatives	1.133	up
MEDP1480	Anserine	Amino acid and Its metabolites	1.130	up
MEDP0050	L-Carnosine	Amino acid and Its metabolites	1.083	up
MEDN1247	1-pyrroline-4-hydroxy-2-carboxylate	Heterocyclic compounds	1.069	up
MEDP1098	Methoxyindoleacetic Acid	Heterocyclic compounds	1.065	up
MEDN0196	Pyrroloquinoline Quinone	Organic acid And Its derivatives	−1.008	down
MEDP1083	Oxypurinol	Nucleotide And Its metabolites	−1.053	down
MEDP1498	8-Azaguanine	Nucleotide And Its metabolites	−1.053	down
MEDP0152	3′-Aenylic Acid	Nucleotide And Its metabolites	−1.083	down
MEDP2428	Cyclo (Ala-Pro)	Amino acid and Its metabolites	−1.105	down
MEDP1947	Ser-Ile	Amino acid and Its metabolites	−1.106	down
MEDP1177	N-Methyl-α-aminoisobutyric acid	Amino acid and Its metabolites	−2.378	down
MEDN0249	Pantothenol	CoEnzyme and vitamins	−2.515	down
MEDP2492	Arg-Ser	Amino acid and Its metabolites	−2.536	down
MEDP2446	cyclo (pro-pro)	Amino acid and Its metabolites	−2.664	down
MEDP1885	Pro-Ile	Amino acid and Its metabolites	−3.697	down
MEDN1441	10-HDoHE	FA	−8.638	down
MEDN0168	Thymidine	Nucleotide And Its metabolites	−9.883	down
MEDP1928	Cyclo (Pro-Val)	Amino acid and Its metabolites	−10.361	down
MEDN1081	12,13-DiHOME	FA	−10.709	down
MEDN1454	15-keto Prostaglandin F2α	FA	−10.855	down
MEDP1834	Glycerol-Tributyrate	GL	−11.939	down
MEDN0448	Pyridoxine 5′-Phosphate	CoEnzyme and vitamins	−14.940	down

**FIGURE 4:**
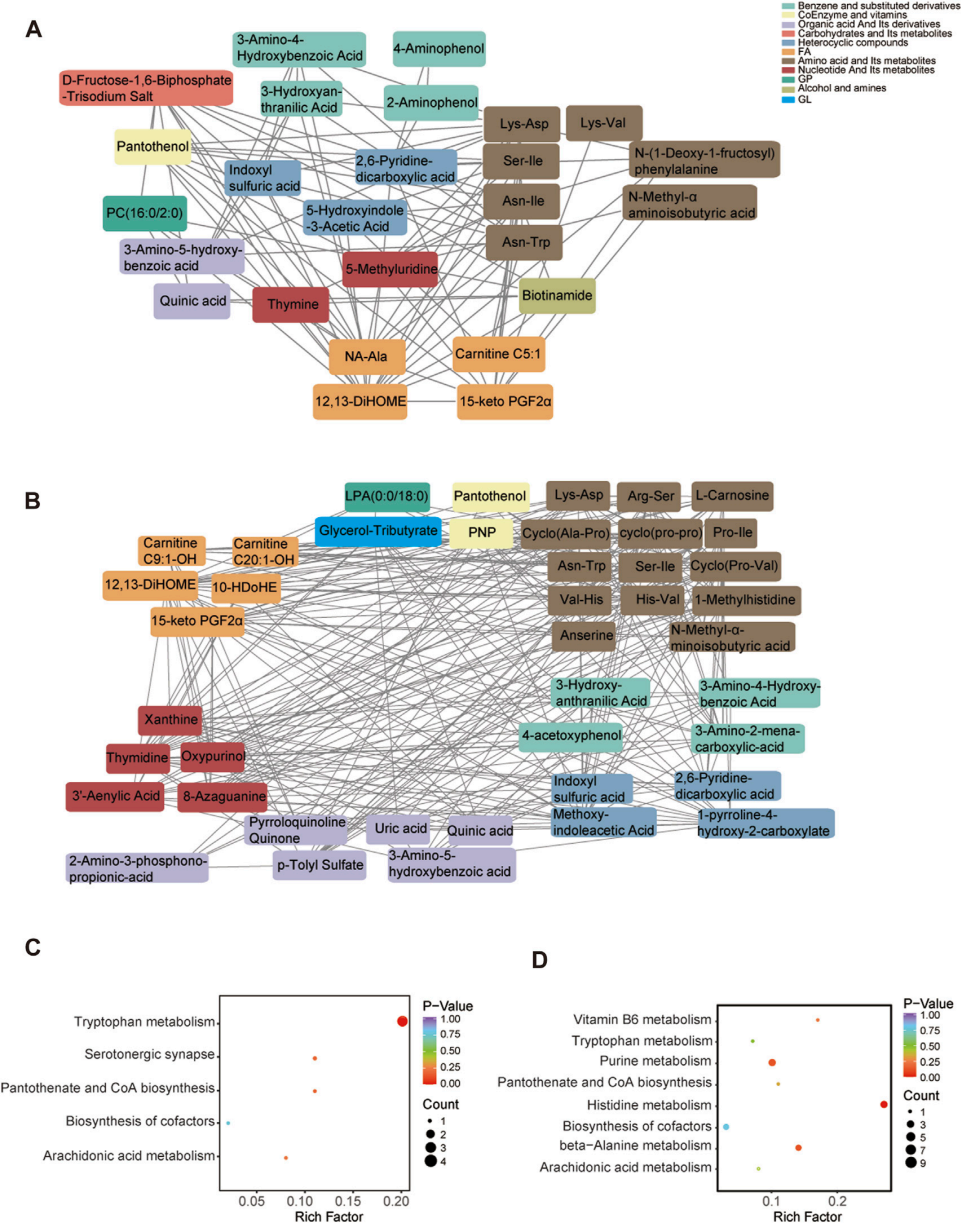
Metabolic correlation network analysis. **(A,B)** Dots represent significantly different metabolites. Differential metabolites with | r | > 0.8 and *p* < 0.05 are selected. Metabolic correlation network of 26 significant differential metabolites based on sham vs. CCI 7 days **(A)**. Metabolic correlation network of 42 significant differential metabolites based on sham vs. CCI 21 days **(B)**. **(C,D)** KEGG pathway analysis of DEMs at CCI 7 days **(C)** and CCI 21 days **(D)**. The comparison of pathway enrichment in the ACC of rats following CCI surgery. The significantly enriched KEGG pathways are shown. The KEGG terms are plotted as the ordinate and the rich factor is plotted as the abscissa. The size of the dots represented the gene number. The color saturation from blue to red indicated *p*-value (Student-Newman-Keuls test, *n* = 4).

To further investigate the regulation of metabolites after peripheral nerve injury, we constructed two correlation networks combining 26 metabolites from sham vs. CCI 7 days and 42 metabolites from sham vs. CCI 21 days. Only the pairs with a Pearson correlation coefficient >0.8 were included in this analysis (Figures 4A, B). The network in Figure 4A (visualized using Cytoscape) included 26 nodes connected by 96 edges. Pairwise correlations between metabolites showed that 54 and 42 pairs of nodes were positively and negatively correlated, respectively. The network in Figure 4B included 42 nodes connected by 291 edges. The pairwise correlations between metabolites showed that 147 and 144 pairs of nodes exhibited positive and negative correlations. Nodes with more connections to other nodes were considered to be important metabolites for the correlation network. As shown in Figures 4A, B, amino acid metabolites had a close connection with other metabolites, indicating that this class of metabolites played an important role in neuropathic pain and pain-related depression.

In addition, to explore the pathways influenced by peripheral nerve injury, the DEMs (including upregulated and downregulated) in sham vs. CCI 7days and sham vs. CCI 21days were annotated according to the pathway types in KEGG (Figures 4C, D). Specifically, tryptophan metabolism, pantothenate and CoA biosynthesis, arachidonic acid metabolism, and biosynthesis of cofactors pathways significantly altered in the rats at CCI 7 days and CCI 21 days compared to that of the sham group. The serotonergic synapse pathway only changed at 7 days after CCI surgery. The vitamin B6 metabolism, purine metabolism, histidine metabolism and beta-alanine metabolism pathways specifically changed at 21 days after CCI surgery. Our metabolome sequencing results showed that the amino acid metabolites changed most significantly in the ACC of rats after peripheral nerve injury, and KEGG analysis also involved several amino acid metabolic pathways such as tryptophan metabolism, histidine metabolism and beta-alanine metabolism. These metabolites may play a role in the development of neuropathic pain and pain-related depression.

### 3.4 Integrated analysis of metabolomics and transcriptomics

**FIGURE 5:**
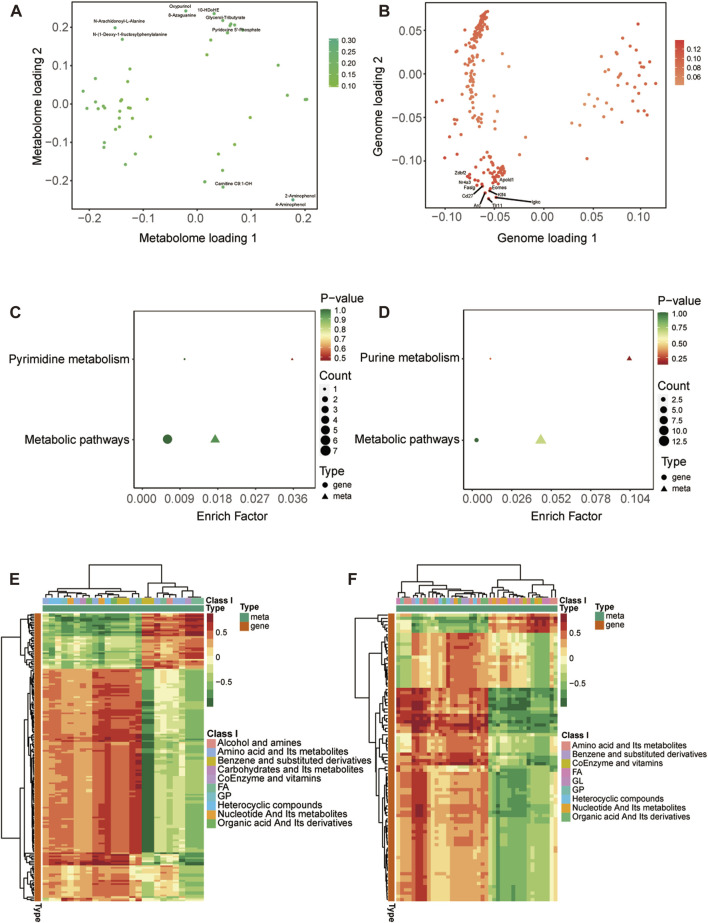
Integrated analysis of metabolomics and transcriptomics. **(A,B)** O2PLS analysis of the sham group and CCI group. Loading 1 represents a one-dimensional loading value and loading 2 represents a two-dimensional loading value. The loading value indicates the explanatory power of the variable in each component, positive or negative loading value indicates a positive or negative correlation with another group, the larger the absolute value of the loading value, the stronger the correlation. The top 10 substances that have a strong influence on the other group are labeled in the metabolomic **(A)** and transcriptomic **(B)** association load maps. **(C,D)** KEGG pathway enrichment of differential genes and metabolites at CCI 7 days **(C)** and CCI 21 days **(D)**. **(E,F)** Heatmap of correlation coefficient matrix between differential metabolites and differential genes with the Pearson correlation coefficient above 0.8. Red represents positive gene-metabolite correlation and green represents negative gene-metabolite correlation. The color labels represent Pearson correlation coefficient values.

We have reported previously the transcriptomic profiles of the ACC in sham group and CCI group rats which suggested that neuropathic pain is initiated and maintained differently by chemokines and their targeting genes (Zhang et al., 2022). Combined with above transcriptome data, we performed conjoint analysis of metabolomics and transcriptomics to screen out the genes and metabolites. To find marker genes and metabolites involved in neuropathic pain and pain-related depression, and to highlight functional associations between these genes and metabolisms, data integration was performed of the other expressed by loading. Figures 5A, B showed the top 10 substances that had a strong influence on the other omics. The top 10 metabolites included NA-Ala, n-(1-deoxy-1-fructosyl) phenylalanine, oxypurinol, glycerol-tributyrate, 10-HDoHE, 8-azaguanine, PNP, Carnitine C9:1-OH, 2-aminophenol and 4-aminophenol. The top 10 genes included zinc finger DBF-type containing 2 (Zdbf2), nuclear receptor subfamily 4 group A member 3 (Nr4a3), apolipoprotein L domain containing 1 (Apold1), Fas ligand (Faslg), Cd27 molecule (Cd27), eomesodermin (Eomes), KLF transcription factor 4 (Klf4), activity regulated cytoskeletal-associated protein (Arc), toll-like receptor 11 (Tlr11) and immunoglobulin kappa constant (Igkc). The KEGG enrichment analysis showed the co-enrichment pathways of DEMs and DEGs (Figures 5C, D). As shown in Figures 5C, D, the most remarkably enriched pathway was the metabolic pathway, which is enriched to 5 metabolites and 7 genes at CCI 7 days, 13 metabolites and 3 genes at CCI 21 days, including cofactor PNP, tryptophan metabolites 3-hydroxy anthranilic acid, 5-hydroxyindole-3-acetic acid and 2-aminophenol, histone metabolites L-carnosine, anserine and 1-methylhistidine, purine metabolites xanthine and uric acid and pyrimidine metabolite thymidine.

The cor program in R was used to calculate the Pearson correlation coefficients (PCCs) between genes and metabolites. We selected the genes and metabolites which had a PCC greater than 0.8 and created a clustered heatmap (Figures 5E, F). In Figure 5E, the clustered heatmap indicated that the differential metabolites associated with differential genes could be sorted into 10 categories, among which amino acid metabolites and fatty acyls were the largest. In Figure 5F, the clustering heat map revealed that the differential metabolites related to differential genes could be categorized into 9 groups, with amino acid metabolites and fatty acyls being the largest group.

### 3.5 Regulating network of DEMs and DEGs and verification of gene expression levels

**FIGURE 6:**
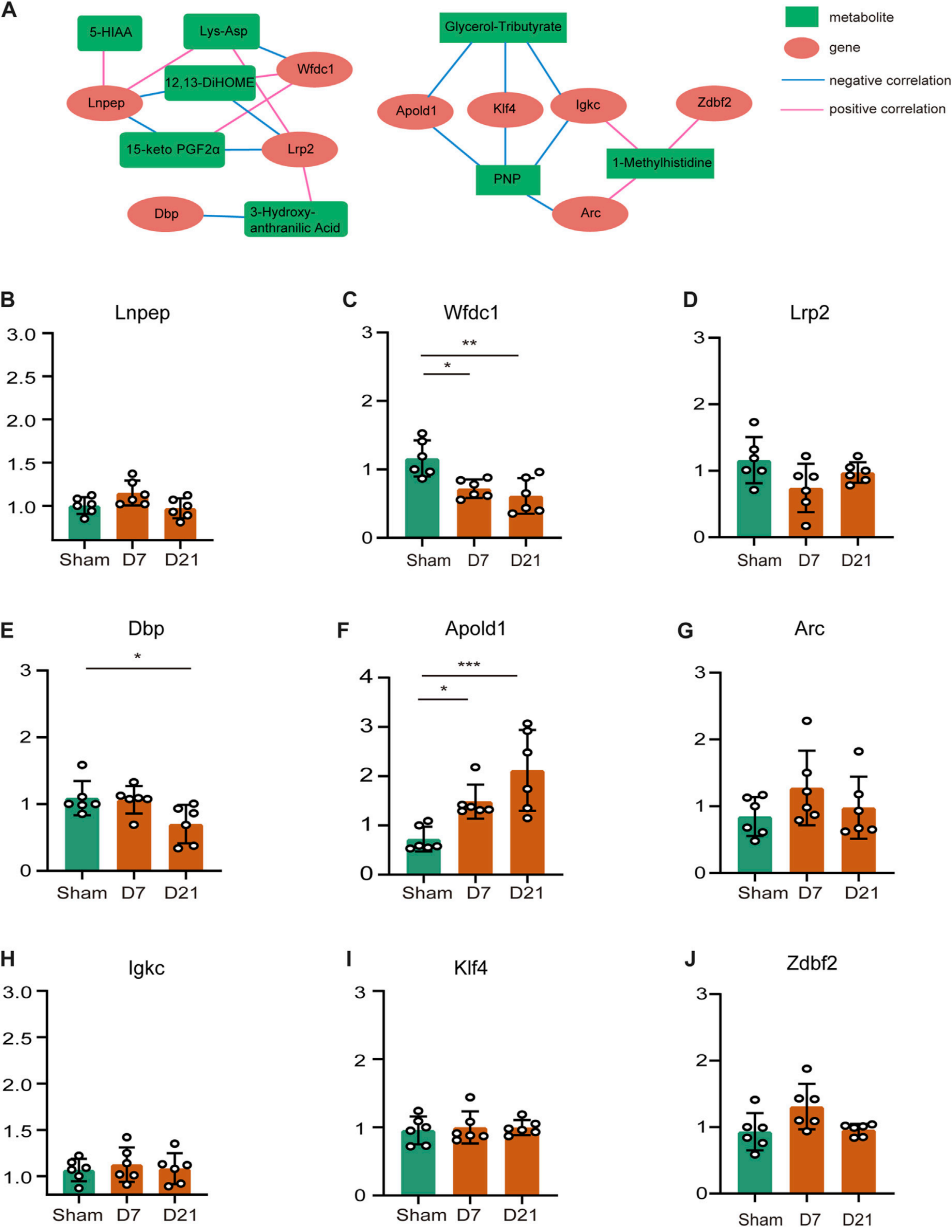
Regulating network of DEMs and DEGs and verification of gene expression levels. **(A)** Regulating network between the genes and metabolites. Metabolites are marked with green squares and genes are marked with red circles. Pink lines represent positive correlations and blue lines represent negative correlations. **(B–J)** Genes that appeared on the network were measured by qRT-PCR to ensure mRNA expression. (*p < 0.05, **p < 0.01, ***p < 0.001, *n* = 6, one-way ANOVA followed by Tukey’s multiple comparison test.)

We focused on the effects of amino acid metabolites and fatty acyls on neuropathic pain, pain-related depression and their possible mechanisms. We chose Lys-Asp, PNP, 15-keto-PGF2α, glycerol-tributyrate, 12,13-diHOME, histidine-related and tryptophan-related metabolites for the analysis of the regulation network between the differential genes and metabolites after peripheral nerve injury. Positive correlations could result from two metabolites originating from a common precursor or occurring adjacent to each other in a metabolic pathway. Negative correlations may indicate that one metabolite generates the other directly or indirectly. Figure 6A showed the regulatory network of differential metabolites after peripheral nerve injury. We subsequently verified the expression of these genes by RT-qPCR (Figures 6B–J) and found that Apold1 elevated and WAP four-disulfide core domain 1 (Wfdc1) downregulated both at CCI 7 days and CCI 21 days. According to the regulating network of DEMs and DEGs, Apold1 was negatively correlated with PNP and glycerol-tributyrate. Wfdc1 was positively associated with 12,13-diHOME and 15-keto-PGF2α, and negatively associated with Lys-Asp. In summary, further study are required to validate the relationship involving the mechanism of peripheral nerve injury resulting in neuropathic pain, pain-related depression, and these metabolites and genes.

## 4 Discussion

The purpose of this study was to investigate the metabolic changes and potential gene associations in a rat model of CCI using metabolomic and transcriptomic analysis. We observed significant alterations in amino acid metabolites and fatty acyls, suggesting their potential roles in neuropathic pain and pain-related depression caused by peripheral nerve injury. Combined analysis with our previously published transcriptome data indicated that some genes were connected with these metabolites. Apold1 was negatively correlated with PNP. Wfdc1 was positively associated with 12,13-diHOME acid and 15-keto PGF2α, and negatively associated with Lys-Asp. This study serves as reference for further research of the neuropathic pain and pain-related depression caused by peripheral nerve injury, which suggests that the changes of amino acid metabolites and fatty acyls may play important roles. However, the effects and mechanisms of the metabolites and genes need to be further validated.

In our untargeted metabolomics analysis of the CCI model, we observed substantial changes in amino acid metabolites and fatty acyls. These findings are consistent with previous studies highlighting the involvement of these pathways in neuropathic pain (Larson et al., 2000; Hua et al., 2021) and negative emotions (Menzies et al., 2021). Our KEGG analysis demonstrated pathways which have been reported to function in the initiation, progression, and therapeutic progress of neuropathic pain and pain-related depression. Specifically, tryptophan metabolites were found to have both neurotoxic and neuroprotective effects in response to inflammation and play a crucial role in chronic pain and depression (Athnaiel et al., 2022). Indole sulfate, a metabolic product of tryptophan (Huć et al., 2018), has been revealed to induce oxidative stress and inflammation in CNS cells (Adesso et al., 2017), leading to neuroinflammation and brain dysfunction (Ohtsuki et al., 2002; Watanabe et al., 2014). Purine metabolism has been identified as the pathway associated with major depressive disorder in young patients (Zhou et al., 2019). Our results showed a significant increase in indole sulfate, 5-hydroxy indole-3-acetic acid, 3-hydroxy anthranilic acid, xanthine and uric acid in the ACC after peripheral nerve injury, which was consistent with most studies. Furthermore, deficiencies in pantothenic acid and vitamin B6 have been associated with pro-inflammatory responses and various symptoms, including arthritic pain, depression, and insomnia (Ueland et al., 2017; Menzies et al., 2021; Ghavidel-Parsa et al., 2022). In line with this, our results demonstrated decreased levels of panthenol and PNP (a form of vitamin B6) in the ACC following peripheral nerve injury. Our metabolomics results highlight roles for tryptophan metabolism, purine metabolism, and vitamin B6 metabolism in neuropathic pain and pain-related depression, suggesting that we should focus on these metabolic pathways. However, not all metabolites changed as reported in the literature. We observed a significant reduction in 15-keto-prostaglandin F2α and 12,13-DiHOME, which were expected to be upregulated according to the published studies (Muratani et al., 2003; Zimmer et al., 2018). The above results suggest that it is difficult to figure out the changes occurring in rat ACC by metabolomics alone, so we combined previously published transcriptomic data for joint analysis.

When integrating our metabolomic findings with previously published transcriptome data, we observed consistent changes in metabolites associated with inflammation and oxidative stress, including xanthine (Stover et al., 1997), uric acid (Stover et al., 1997), 15-keto-PGF2α (Saenger et al., 2007), 12,13-diHOME (Zimmer et al., 2018), L-carnosine (Fleisher-Berkovich et al., 2009), anserine (Song et al., 2014), glyceryl tributyrate (Segain et al., 2000; Vinolo et al., 2012; Xin et al., 2018), etc. These findings align with transcriptomic studies (Zhang et al., 2022), reporting the differential genes in ACC of rats after peripheral nerve injury were involved in biological processes such as “immune system processes”, “defense responses”, “regulation of immune system processes”, and “regulation of cellular processes”. We confirmed that Apold1 elevated and Wfdc1 downregulated both at CCI 7 days and CCI 21 days. Apold1 and Wfdc1 were generally located in endothelial cells and regulated endothelial cell signaling and vascular function (Regard et al., 2004; Zhu et al., 2021). There are also some reports indicating Apold1 may be expressed in neurons and astrocytes and Wfdc1 could be expressed in neurons (Koshimizu et al., 2021). It has been reported that heightened levels of Apold1 have been observed in the prefrontal cortex, cerebellum, and hippocampus of stressed mice (Roszkowski et al., 2014) and Wfdc1 had the anti-inflammatory function (Reading et al., 2012). Nevertheless, the relationship between the two genes and pain has not yet been shown. Our study predicted that Apold1 was negatively correlated with PNP and glycerol-tributyrate. Wfdc1 was positively associated with 12,13-diHOME and 15-keto-PGF2α, and negatively associated with Lys-Asp. We should pay close attention to these genes and their associated metabolites when exploring potential mechanisms of neuropathic pain and pain-related depression after peripheral nerve injury.

This study is the first to probe the molecular changes of ACC in rats with neuropathic pain and pain-related depression both metabolically and transcriptionally. The present study has some limitations that need to be acknowledged. Firstly, the model used in this study is limited to CCI, which may not fully represent all types of neuropathic pain. Additionally, the brain region examined in this study is restricted to the ACC, and it is important to consider that other brain regions may also play significant roles in neuropathic pain and pain-related depression. Importantly, it should be noted that our study provides correlative evidence, and no direct manipulation of the molecular changes or assessment of their impact on behavior was conducted. The observed molecular changes may co-occur due to the injury and neuroinflammation rather than being causal factors. Nonetheless, our findings shed light on the potential involvement of these metabolites and genes in peripheral nerve injury-induced neuropathic pain and pain-related depression, offering valuable insights for future research and therapeutic approaches.

## Data Availability

The datasets presented in this study can be found in online repositories. The names of the repository/repositories and accession number(s) can be found in the article/supplementary material.
